# Senolytics prevent caveolar Ca_V_3.2‐RyR axis malfunction in old vascular smooth muscle

**DOI:** 10.1111/acel.14002

**Published:** 2023-10-14

**Authors:** Jie Lin, Weiming Guo, Qingtian Luo, Qingping Zhang, Teng Wan, Changyu Jiang, Yuanchun Ye, Haihuan Lin, Gang Fan

**Affiliations:** ^1^ Cardiology Department The first Affiliated Hospital of Wenzhou Medical University Wenzhou China; ^2^ Sports Medicine Center Huazhong University of Science and Technology Union Shenzhen Hospital, the 6th affiliated Hospital of Shenzhen University Medical School Shenzhen China; ^3^ Department of Gastroenterology Huazhong University of Science and Technology Union Shenzhen Hospital, the 6th affiliated Hospital of Shenzhen University Medical School Shenzhen China; ^4^ Neurology Department Huazhong University of Science and Technology Union Shenzhen Hospital, the 6th affiliated Hospital of Shenzhen University Medical School Shenzhen China; ^5^ Department of Pain Medicine and Shenzhen Municipal Key Laboratory for Pain Medicine Huazhong University of Science and Technology Union Shenzhen Hospital, the 6th affiliated Hospital of Shenzhen University Medical School Shenzhen China; ^6^ Quanzhou First Hospital Affiliated to Fujian Medical University Quanzhou Fujian Province China; ^7^ Urology department, Huazhong University of Science and Technology Union Shenzhen Hospital the 6th affiliated Hospital of Shenzhen University Medical School Shenzhen China; ^8^ Hunan Cancer Hospital, the Affiliated Cancer Hospital of Xiangya School of Medicine Central South University Changsha China

**Keywords:** aging, calcium sparks, caveolae, senolytics, T‐type calcium channels, vascular smooth muscle

## Abstract

Aging is a major risk factor for cardiovascular diseases. Our previous studies demonstrate that aging impairs the caveolar T‐type Ca_V_3.2‐RyR axis for extracellular Ca^2+^ influx to trigger Ca^2+^ sparks in vascular smooth muscle cells (VSMCs). We hypothesize that the administration of senolytics, which can selectively clear senescent cells, could preserve the caveolar Ca_V_3.2‐RyR axis in aging VSMCs. In this study, 10‐month‐old mice were administered the senolytics cocktail consisting of dasatinib (5 mg/kg) and quercetin (50 mg/kg) or vehicle bi‐weekly for 4 months. Using VSMCs from mouse mesenteric arteries, we found that Ca^2+^ sparks were diminished after caveolae disruption by methyl‐β‐cyclodextrin (10 mM) in cells from D + Q treated but not vehicle‐treated 14‐month‐old mice. D + Q treatment promoted the expression of Ca_V_3.2 in 14‐month‐old mesenteric arteries. Structural analysis using electron tomography and immunofluorescence staining revealed the remodeling of caveolae and co‐localization of Ca_V_3.2‐Cav‐1 in D + Q treatment aged mesenteric arteries. In keeping with theoretical observations, Ca_v_3.2 channel inhibition by Ni^2+^ (50 μM) suppressed Ca^2+^ in VSMCs from the D + Q group, with no effect observed in vehicle‐treated arteries. Our study provides evidence that age‐related caveolar Ca_V_3.2‐RyR axis malfunction can be alleviated by pharmaceutical intervention targeting cellular senescence. Our findings support the potential of senolytics for ameliorating age‐associated cardiovascular disease.

## Graphical abstract

The use of a senolytic cocktail, consisting of dasatinib and quercetin, could potentially improve the functionality of aging arteries by targeting and eliminating senescent cells. This treatment may lead to enhancements in the expression of Ca_V_3.2 channels and caveolae remodeling, resulting in an improved Ca_V_3.2‐RyR axis and subsequent increase in Ca^2+^ spark generation. Utilizing this senescent cell clearance approach has the potential to be a promising therapeutic method in enhancing vascular function among elderly individuals.

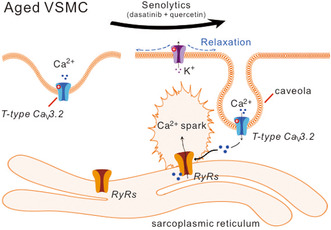

**Abbreviations**
BKca: large‐conductance Ca2+‐sensitive K+
Cav‐1: caveolin‐1
D: dasatinib
GSEA: gene set enrichment analysis
Q: quercetin
SASP: senescence‐associated secretory phenotype
SR: sarcoplasmic reticulum
STOCs: spontaneous transient outward K+ currents
VSMCs: vascular smooth muscle cells

## 1 INTRODUCTION

Aging is a major cardiovascular risk factor that is associated with impairment of vascular smooth muscle cells (VSMCs) and endothelial function, which may potentially lead to cardiovascular disease (Ungvari et al., 2018). During aging, several signaling modalities are altered along with vascular remodeling (Zhou et al., 1998). An indirect mechanism involving Ca^2+^ release events (known as Ca^2+^ sparks) has been identified to attenuate arterial tone and limit excessive vasoconstriction. T‐type Ca_V_3.2 channels, which are localized in caveolae, mediate Ca^2+^ influx and stimulates the cytosolic domain of ryanodine receptors (RyRs) to induce Ca^2+^ release from the sarcoplasmic reticulum (SR) in the form of Ca^2+^ sparks, and thus opens numerous large‐conductance Ca^2+^‐sensitive K^+^ (BK_Ca_) channels causing spontaneous transient outward K^+^ currents (STOCs). As a result, Ca^2+^ spark–BK_Ca_ channel coupling induces VSMCs hyperpolarization and the attenuation of arterial constriction (Fan et al., 2018). The localization of Ca_V_3.2 in caveolae closed to RyRs is crucial for triggering Ca^2+^ sparks. Advanced age has been found to alter the composition of lipid rafts, and the morphology of caveolae in SMCs (Lowalekar et al., 2012; Ratajczak et al., 2003). With aging, caveolar Ca_V_3.2 channels are impaired in triggering Ca^2+^ sparks in VSMCs in aged mice (12–14 months). Furthermore, there was no difference in the myogenic tone between aged mesenteric arteries from Ca_V_3.2 Ca^2+^ channel deficient (Ca_V_3.2^−/−^) and wild‐type mice, despite an enhanced tone in young Ca_V_3.2^−/−^ mice compared to controls (Mikkelsen et al., 2016). The malfunction of T‐type Ca_V_3.2 channels may be caused due to age‐related ultrastructural changes of caveolae, which are not closely situated to RyRs for extracellular Ca^2+^‐influx through T‐type channels to trigger Ca^2+^‐sparks (Fan et al., 2020).

There is strong experimental and clinical evidence suggesting that targeting cellular senescence could delay the aging process and alleviate age‐related diseases (Hickson et al., 2019; Justice et al., 2019; Kirkland & Tchkonia, 2020; Zhu et al., 2015). Senolytics, which can specifically kill senescent cells, show promise in collectively delaying multiple diseases. D + Q, a combination treatment of dasatinib (D), a tyrosine kinase inhibitor, with quercetin (Q) has been best studied of senolytics in cardiovascular diseases, to improve ventricular function and vasomotor function (Zhu et al., 2015). Caveolin‐1 (Cav‐1) is the major coat protein essential for caveolae formation. Dasatinib, one of the most potent TKIs, interferes with the activity of several kinases of the Src family, targets Cav‐1 contributing to cancer treatment (Ortiz et al., 2020). Additionally, the antioxidant agent quercetin could prevent the pro‐inflammatory responses and oxidative stress‐induced senescent phenotype by regulating caveolae (Kondo‐Kawai et al., 2021). D + Q present highly clinical translational potention since both drugs have approved for use in humans and have demonstrated relative safety with oral administration, its anti‐aging protective effect has been observed and appeared promising in patients with idiopathic pulmonary fibrosis (Justice et al., 2019), diabetic kidney disease (Hickson et al., 2019), and Alzheimer's disease (Kirkland & Tchkonia, 2020). However, the effect of D + Q on aged VSMCs remains unknown. In this study, we investigated the efficacy of the senolytics cocktail dasatinib plus quercetin (D + Q) on the caveolar Ca_V_3.2‐RyR axis in VSMCs in middle‐aged artery (14‐month‐old).

## 2 SENOLYTICS ON CAVEOLAE‐RYR COUPLING

**FIGURE 1:**
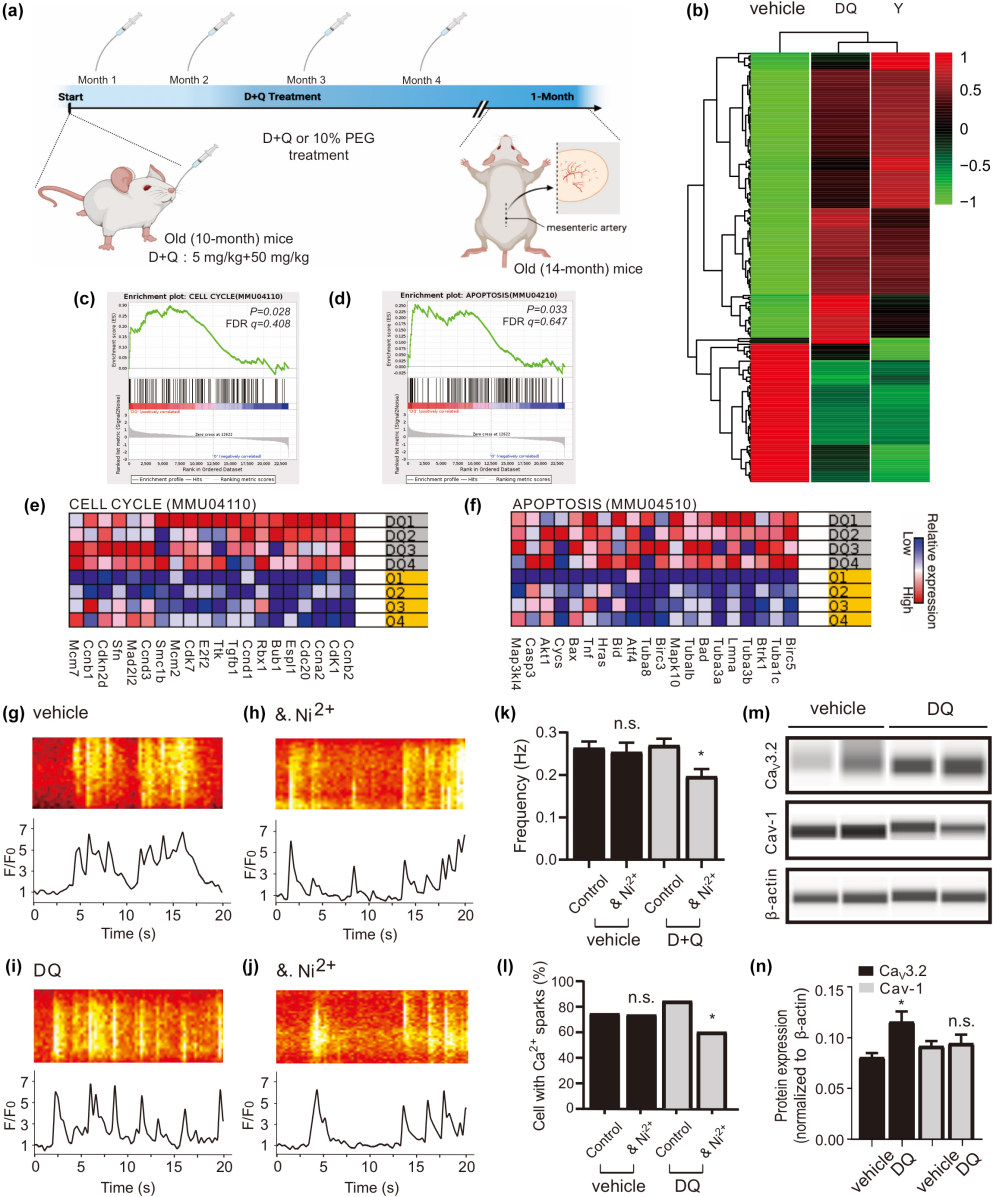
D + Q turnover caveolar Ca^2+^ sparks in aged mesenteric VSMCs. (a), experimental design of senolytics administration is shown. (D, dasatinib; Q, quercetin; PEG, polyethylene glycol). (b), heat map of RNA‐seq data of mesenteric artery from young, vehicle‐, and D + Q‐treatment mice (*n* = 4 samples for each group). (c–f) Shown are gene set enrichment analyses (c, d) and heat maps for the top 20 up‐regulated genes for CELL CYCLE (e) and APOPTOSIS (f) in D + Q compared with vehicle group. FDR, false discovery rate. (g), Ca^2+^ fluorescence line scan images of a Fluo‐4‐AM–loaded VSMC from a middle‐aged mouse and the time course of Ca^2+^ fluorescence changes. (h), same as (g) but in the presence of methyl‐β‐cyclodextrin (dextrin, 10 mM, 90 min at room temperature). (i), same as (g) but in the cell from a D + Q‐treated aged mouse. (j), same as (i) but in the presence of methyl‐β‐cyclodextrin. (k–l), summary of the results. Ca^2+^ spark frequency (e) and fraction of cells producing Ca^2+^ sparks (f) in VSMCs from aged mice (*n* = 134), in VSMCs from aged mice incubated with methyl‐β‐cyclodextrin (*n* = 95), in VSMCs from D + Q treated aged mice (*n* = 175), and in VSMCs from D + Q treated aged mice incubated with methyl‐β‐cyclodextrin (*n* = 175). Cells were isolated from 4 mice in each group. VSMC, vascular smooth muscle cell. *p < 0.05; n.s., not significant. (m), western blot analysis of Ca_V_3.2, Caveolin‐1 proteins in mesenteric arteries of aged versus D + Q mice. (n), quantification of western blot results. Western blot results were analyzed from 8 mice in each group. *p < 0.05; n.s., not significant; Cav‐1, Caveolin‐1.

To investigate the impact of D + Q on the aging mesenteric artery, we analyzed RNA sequencing data from a mesenteric artery of 14‐month‐old mice treated with either vehicle or D + Q (Figure 1a). We identified a total of 855 genes that were differentially expressed between young, senolytic‐, and vehicle‐treated arteries of 14‐month‐old mice (Figure 1b). Using Gene Set Enrichment Analysis (GSEA), we found numerous signaling molecules and genes implicated in D + Q treatment responses, including CELL CYCLE (mmu04110), and APOPTOSIS (mmu04510) in the artery (Figure 1c–f). The most harmful senescent cells are resistant to apoptosis and have up‐regulated anti‐apoptotic pathways that protect them from their own inflammatory senescence‐associated secretory phenotype (SASP). Consequently, eliminating these cells through the body's natural mechanisms for removing damaged or unwanted cells becomes a challenge (Hu et al., 2022). Senolytics have been proposed as a means of inducing apoptosis in senescent cells by inhibiting TAF^+^, p16INK4A, BCL‐xL, PI3KCD, p21, PAI1, and PAI2 (Xu et al., 2018). This selective clearance of harmful senescent cells has the potential to reduce the negative effects of ageing microenvironment and improve tissue function in both aging and age‐related diseases. Importantly, D + Q induces apoptosis strictly in senescent cells, rather than non‐senescent controls observed in vitro (Xu et al., 2018). In our study, D + Q did not alter hallmark SASP genes (Figure S1a), which can be explained by the less expression of SASP in early vascular aging. Taken together, these findings suggest that D + Q has a senolytic activity by disabling the senescence‐associated anti‐apoptotic pathways (SCAPs), which typically protect senescent arteries in early vascular aging.

To ascertain the contribution of D + Q in regulating the caveolae‐RyR coupling, we conducted line‐scan Ca^2+^ measurements on isolated arteries from 14‐month‐old mice. The contribution of caveolae in Ca^2+^ spark generation was assessed in vehicle‐ and D + Q‐treated VSMCs using methyl‐β‐cyclodextrin (10 mM), a cholesterol‐depleting drug known to disturb caveolae and inhibit a significant fraction of Ca^2+^ sparks in VSMCs (Fan et al., 2018). In accordance with our previous data (Fan et al., 2020), we observed that methyl‐β‐cyclodextrin did not affect Ca^2+^ spark generation in 14‐month‐old VSMCs; however, methyl‐β‐cyclodextrin decreased the frequency of Ca^2+^ spark and the fraction of cells with sparks after D + Q treatment (Figure 1g–l), consistent with the data that D + Q treatment improved Ca^2+^ spark generation in 14‐month‐old VSMCs (Figure S2a). These data suggest that D + Q may restore the coupling between caveolae‐RyR and Ca^2+^ sparks generation in middle‐aged VSMCs. To address whether the improved caveolae function in generating Ca^2+^ sparks in aged VSMCs relies on increased protein expression, we analyzed the level of Cav‐1 and Ca_V_3.2 proteins in arteries from 14‐month‐old mice treated with D + Q or vehicle. Interestingly, D + Q treatment was able to promote the expression of Ca_V_3.2 but not Cav‐1 in aged arteries (Figure 1m,n, also see Figure S1b,c).

## 3 SENOLYTICS ON VSMC CAVEOLAE REMODELING AND CA_V_3.2‐RYR AXIS

**FIGURE 2:**
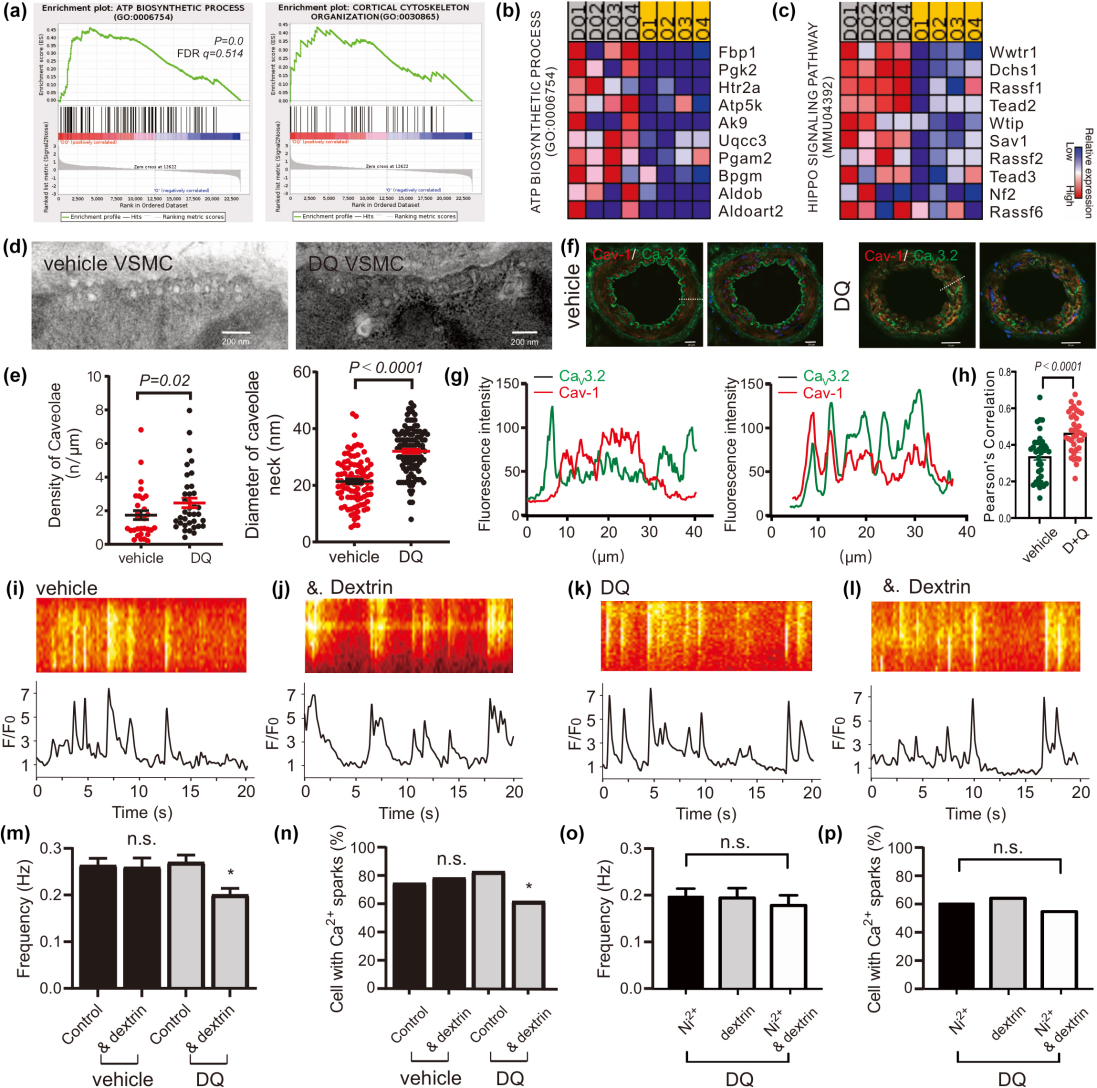
D + Q promote caveolae remodeling and Ca_V_3.2‐Cav‐1 co‐localization to rescue caveolar Ca_V_3.2‐RyR axis in aged VSMC. (a–c), shown are gene set enrichment analyses (a) and heat maps for the top 10 up‐regulated genes (b, c) for ATP BIOSYNTHETIC PROCESS and MICROTUBULE CYTOSKELETON ORGANIZATION in D + Q compared with vehicle group. (d, e), electron microscopy image of a vehicle and a D + Q treated VSMC, and summary of the results. Caveolae density, diameter of caveolae neck in VSMCs from vehicle‐ versus D + Q‐treated mice (four mice in each group). (f‐g), confocal immunofluorescence images and the line course of fluorescence changes. Immunofluorescently labeled with Ca_V_3.2 (green) and Cav‐1 (red) in mesenteric arteries from vehicle‐ and D + Q‐treated mice. Bar, 20 μm. (h), Pearson's correlation coefficients for colocalization assays. The plot shows Pearson's correlation coefficients for the colocalization analysis (*n* = 30–40 arteries). The Kruskal–Wallis *H* test was used for calculating statistical differences. Arteries were isolated from four mice in each group. (i), Ca^2+^ fluorescence line scan images of a Fluo‐4‐AM–loaded VSMC from a middle aged mouse and the time course of Ca^2+^ fluorescence changes. (j), same as (h) but in the presence of Ni^2+^ (50 μM). (k), same as (h) but in the cell from a D + Q treated aged mouse. (l), same as (j) but in the presence of Ni^2+^. (m–p), summary of the results. Ca^2+^ spark frequency (m) and fraction of cells producing Ca^2+^ sparks (n) in VSMCs from aged mice (*n* = 124), in VSMCs from aged mice incubated with Ni^2+^ (*n* = 109), in VSMCs from D + Q‐treated aged mice (*n* = 176), and in VSMCs from D + Q‐treated aged mice incubated with Ni^2+^ (*n* = 115). Ca^2+^ spark frequency (o) and fraction of cells producing Ca^2+^ sparks (p) in VSMCs from D + Q treated aged mice incubated with Ni^2+^ (*n* = 112), with methyl‐β‐cyclodextrin (*n* = 108), as well as Ni^2+^+methyl‐β‐cyclodextrin (*n* = 96). Cells were isolated from four mice in each group. VSMC, vascular smooth muscle cell. *p < 0.05; n.s., not significant.

We hypothesize that the rescue of caveolar Ca^2+^ spark generation in middle‐aged VSMCs by D + Q treatment could result from the remodeling of caveolae, where Ca_V_3.2 channels reside to drive RyR‐mediated Ca^2+^ sparks (Fan et al., 2018). GSEA reveals the ATP BIOSYNTHETIC PROCESS (GO: 0006754) and MICROTUBULE CYTOSKELETON ORGANIZATION (GO: 0000226) in the responses to D + Q treatment in 14‐month‐old arteries (Figure 2a–c), consist the idea that the cell cytoskeleton and microtubules promote recycling of caveolae contributing the distribution of caveolae as well as trafficking at the plasma membrane (Echarri et al., 2012). Moreover, intrinsic ATPases involved in membrane remodeling in the endosomal system are essential in restricting caveolae dynamics in cells. The EH‐domain–containing protein 2 (EHD2), a dynamin‐related ATPase, was demonstrated to regulate the stability and turnover of caveolae. The structure of caveolae on the cell membrane is closely associated with their recycling and stability. Previous studies have shown that EHD2 plays a crucial role in regulating the stability of caveolae, as see our previous study (Fan et al., 2020). Therefore, in this study, we investigated the ultrastructure of caveolae in VSMCs treated with either vehicle or D + Q. Our results showed that the density of caveolae and the diameter of the caveolae neck were higher in D + Q‐treated VSMCs compared to the vehicle group cells (Figure 2d,e). To determine whether D + Q treatment enhances Ca_V_3.2 channel caveolar localization in middle‐aged VSMCs, we prepared arteries from 14‐month‐old mice and subjected them to immunofluorescence staining to identify Ca_V_3.2 and Cav‐1. Our findings show that Ca_V_3.2 is more co‐localized with Cav‐1 in D + Q treated arteries than in the vehicle group (Figure 2f–h, also see Figure S1d‐e).

We confirmed these results by measuring the contribution of Ca_V_3.2 to Ca^2+^ spark generation in VSMCs from D + Q‐treated mice. We found the T‐type Ca_V_3.2 channel blocker Ni^2+^ decreased Ca^2+^ spark frequency and the fraction of cells with sparks in D + Q‐treated VSMCs, while it failed to decrease Ca^2+^ spark events in the vehicle group (Fan et al., 2020; Figure 2i–n). The application of Ni^2+^ in the mesenteric VSMCs that were previously treated with methyl‐β‐cyclodextrin yielded no additional decrease in Ca^2+^ spark frequency or the proportion of cells exhibiting firing activity (Figure 2o,p). We next measured BK_Ca_ channel currents activated by Ca^2+^ sparks (STOCs) in aged and D + Q‐treated VSMCs. STOCs were measured in the presence of Ni^2+^ or not. The holding potential was set to −40 mV, a physiological membrane potential that should drive T‐type Ca^2+^ channel‐mediated Ca^2+^ sparks, enabling the activation of BK_Ca_ channels. We found that Ni^2+^ blocked STOCs in cells from D + Q‐treated mice but not in cells from aged mice (Figure S1f–i). In order to rule out the effects of Ca_V_1.2 channels on Ca^2+^ sparks generation, we utilized 200 μM Cd^2+^ before probing Ni^2+^ effects on Ca^2+^ sparks in aged vessels (Fan et al., 2020). Similar results were observed for Ni^2+^ on Ca^2+^ spark generation after silencing Ca_V_1.2 channels in D + Q‐treated old VSMCs but not in the control group (Figure S2b,c). These finding are consistent with previous studies indicating that senescent cell clearance by D + Q would improve VSMC relaxation and alleviates vasomotor dysfunction in naturally aging mice (Roos et al., 2016; Zhu et al., 2015). Overall, D + Q appears to contribute to the up‐regulation of Ca_V_3.2 expression and caveolae remodeling, both of which may play a role in the observed effects of the vascular T‐type Ca_V_3.2‐RyR axis on Ca^2+^ sparks generation in aged VSMCs.

## 4 IN SUMMARY

Ca_V_3.2 channels in caveolar microdomains co‐localize with RyR to initiate Ca^2+^ sparks and activate BK_Ca_ channels to drive a feedback response on vascular tone. However, this mechanism of Ca^2+^ spark generation is influenced by age. Our study demonstrates that administration of D + Q could rescue senescent arteries, improve the expression of Ca_V_3.2 channels and caveolae remodeling, thereby enhancing caveolar Ca_V_3.2‐RyR axis on Ca^2+^ spark generation in aging. Senescent cell clearance could be a promising therapeutic approach to enhancing vascular function among the elderly.

## 5 EXPERIMENTAL PROCEDURES

### 5.1 Mice

In this study, young (12–14 weeks)‐, middle aged (vehicle‐treated) (14 months)‐, D + Q‐treated aged (14 months)‐male mice were used. Mice were maintained at the breeding facility of the Animal Center of Huazhong University of Science and Technology Union Shenzhen Hospital in individually ventilated cages under standardized conditions that included a 12‐h dark–light cycle and free access to standard chow, and drinking water. All mice were deeply anaesthetized by inhalation of isoflurane until cessation of breathing, then killed by cervical dislocation and the mesentery arteries removed. Experiments were performed on the same day with arteries from litter‐matched old versus D + Q mice. All animal protocols were approved by the local animal care committee of Huazhong University of Science and Technology Union Shenzhen Hospital. There are no ethical concerns.

### 5.2 Senolytics treatment

Mice were administered a senolytic cocktail containing 5 mg/kg dasatinib (Selleck Chemicals, S1021) and 50 mg/kg quercetin (Sigma‐Aldrich, Q4951) as described previously (Zhou et al., 2021). Briefly, Dasatinib and quercetin were dissolved in 10% polyethylene glycol 400 (PEG 400; Sigma‐Aldrich, #25322‐68‐3). Mice were gavaged bi‐weekly for 4 months with D + Q or vehicle (10% PEG 400). All mice completed the treatment period and their mean body weights were similar to the nongavage‐fed mice (Figure S1j).

### 5.3 Isolation of arterial vascular smooth muscle cells

Arterial VSMCs from mesenteric arteries were isolated as previously described (Fan et al., 2020). Briefly, arteries were removed and quickly transferred to cold (4°C) oxygenated (95% O_2_–5% CO_2_) physiological salt solution (PSS) of the following composition (mM): 119 NaCl, 4.7 KCl, 1.2 KH_2_PO_4_, 25 NaHCO_3_, 1.2 MgSO_4_, 1.6 CaCl_2_, and 11.1 glucose. The arteries were cleaned, cut into pieces, and placed into a Ca^2+^‐free Hank‘s solution (mM): 55 NaCl, 80 sodium glutamate, 5.6 KCl, 2 MgCl_2_, 1 mg/mL bovine serum albumin (BSA, Sigma), 10 glucose, and 10 HEPES (pH 7.4 with NaOH) containing 0.5 mg/mL papain (Sigma) and 1.0 mg/mL DTT for 37 min at 37°C. The segments were then placed in Hank‘s solution containing 1 mg/mL collagenase (Sigma, type F and H, ratio 30% and 70%) and 0.1 mM CaCl_2_ for 17 min at 37°C. Following several washes in Ca^2+^‐free Hank‘s solution (containing 1 mg/mL BSA), single cells were dispersed from artery segments by gentle triturating. Cells were then stored in the same solution at 4°C.

### 5.4 Ca^2+^ imaging measurements

Ca^2+^ sparks were measured as previously described (Fan et al., 2020). Isolated VSMCs were placed onto glass coverslips and incubated with the Ca^2+^ indicators fluo‐4 AM (10 μM) and pluronic acid (0.005%, w/v) for 60 min at room temperature in Ca^2+^‐free Hanks' solution. After loading, cells were washed with bath solution for 10 min at room temperature. Isolated cells and intact arterial segments were imaged in a bath solution containing (mM): 134 NaCl, 6 KCl, 1 MgCl_2_, 2 CaCl_2_, 10 glucose and 10 HEPES (pH 7.4, NaOH). Images were recorded using confocal microscope (FV3000, Olympus). Images were obtained by illumination with an argon laser at 488 nm, and recording all emitted light above 515 nm. Ca^2+^ spark analyses were performed line‐scan using ImageJ software. The entire area of each image was analyzed to detect Ca^2+^ sparks. Ca^2+^ sparks were defined as local fractional fluorescence increase (*F/F*_*0*_) above the noise level of 1.5. The frequency was calculated as the number of detected sparks divided by the total scan time.

### 5.5 Western blot analysis

The samples were analyzed with a Simple Western assay using the WES™ system (ProteinSimple, Bio‐Techne; Chachoua et al., 2022). The following antibodies were used for the Western analysis: anti‐Ca_V_3.2‐rabbit (Alomone Labs, #ACC‐025, diluted 1:5), anti‐rabbit‐caveolin‐1 (Beyotime, #AF1231, diluted 1:5), and anti‐rabbit‐β‐actin (Abcam, #ab115777, diluted 1:150). The relative amount of each protein was quantified via the peak areas detected in the chemiluminescence electropherogram generated by the Compass for SW software (ProteinSimple), following the default settings. A standard curve based on the serial dilutions of the input was used to estimate the absolute amount of protein in each sample. Finally, the recovery of input for each identified interacting protein was calculated through normalization to the percentage recovery of CTCF. All antibodies were approved of only if they detected the correct bands upon WES/JESS analyses.

### 5.6 Ultrastructure and quantitative assessment of caveolae

Quantitative assessment of caveolae was carried out as previously described (Fan et al., 2020). Isolated VSMCs from mesenteric arteries were dehydrated in a graded series of ethanol and embedded in the PolyBed® 812 resin (Polysciences Europe GmbH), ultrathin sections (60–80 nm) were cut (Leica microsystems), and uranyl acetate and lead citrate staining was performed. Samples were examined at 80 kV with a Zeiss EM 910 electron microscope (Zeiss), and image acquisition was performed with a Quemesa CDD camera and the iTEM software (Emsis GmbH). The density of caveolae was calculated as number of caveolae per micrometer. The diameter of caveolae neck (nm) was determined by using the parallel dimension function of CorelDRAW.

### 5.7 Immunohistostaining of mesenteric arteries for confocal imaging

Mice were anesthetized with 2% ketamine/10% rompun, perfused by 30 mL PBS and 50 mL 4% PFA (Roth, diluted in PBS) and after wards vessels were dissected, and tissue pieces were further fixed for 4 h in 4% PFA, transferred to 15% sucrose (in PBS, Merck) for 4 h and incubated in 30% sucrose overnight. After embedding in TissueTek (Sakura), the tissue is frozen at −80°C and 8 μm sections were obtained in a Leica cryostat at −30°C. For immunostainings, the cryostat sections were incubated with blocking buffer (1% donkey serum/1% TritonX100/PBS), the first antibody was applied overnight at 4°C, and after washing with PBS/1% Tween, the secondary antibody and DAPI were applied for 2 h. Afterward the sections were embedded in ImmoMount (ThermoScientific #9990402). The stained sections were analyzed with confocal microscope (FV3000, Olympus), and images were analyzed by ImageJ. Antibodies: anti‐Ca_V_3.2‐rabbit (Alomone Labs, #ACC‐025), anti‐caveolin‐1‐mouse (Beyotime, #AF0087), DAPI (Sigma, #D9542).

### 5.8 Electrophysiology

Potassium currents were measured in the whole‐cell perforated‐patch mode of the patch‐clamp technique. Patch pipettes (resistance, 1.5–3.5 M) were filled with a solution containing (in mM): 110 potassium aspartate, 30 KCl, 10 NaCl, 1 MgCl_2_ and 0.05 EGTA (pH 7.2). The external bath solution contained (in mM): 134 NaCl, 6 KCl, 1 MgCl_2_, 2 CaCl_2_, 10 glucose and 10 HEPES (pH 7.4); holding potential was −60 mV. Whole cell currents were recorded using an Axopatch 200B amplifier (Axon Instruments/Molecular Devices) or an EPC 7 amplifier (List) at room temperature. Data were digitized at 5 kHz, using a Digidata 1440A digitizer (Axon CNS, Molecular Devices) and pCLAMP software versions 10.1 and 10.2. STOC analysis was performed off‐line using IGOR Pro (WaveMetrics) and Microsoft Excel software. A STOC was identified as a signal with at least three times the BKCa single channel current amplitude.

### 5.9 RNA isolation and RNA sequencing

Mesenteric arteries from vehicle‐ and D + Q‐treated mice (*n* = 4/group) were used to isolate RNA with the Qiagen miRNeasy® Mini Kit (Hilden, Germany). RNA concentration was determined by utilizing a Nanodrop 2000 (Thermo Fisher) and RNA integrity was quantified using an Agilent Bioanalyzer 2100 (Agilent Technologies, Santa Clara, CA). High‐quality RNA (RIN >8.0) of 500 nanograms was then sent to Novogene for sequencing. RNAseq analyses were carried out using Partek® Flow® software, v10.0. The default QA/QC tool was employed for pre‐alignment quality control. The splice‐aware program STAR (v2.7.8a) was used for aligning sequencing reads to the mouse genome (GRCm39). Gene counts were quantified using Partek E/M against transcriptome release 103 with a minimum expression cutoff of 10 counts to filter out low expression genes. Differential gene expression was examined by using DESeq2 (v3.5) with FDR <0.05.

### 5.10 Materials

Fluo‐4‐AM was purchased from Molecular Probes (Thermo Fisher Scientific, #F14201). All salts and other drugs were obtained from Sigma‐Aldrich or Merck. In cases where DMSO was used as a solvent, the maximal DMSO concentration after application did not exceed 0.5%.

### 5.11 Statistics

Data are presented as mean ± SEM. Statistically significant differences in mean values were determined by Student's unpaired *t* test or one‐way analysis of variance (ANOVA) or Mann–Whitney *U* test. *p* < 0.05 were considered statistically significant; “*n*” represents the number of cells.

## AUTHOR CONTRIBUTIONS

All authors were responsible for interpretation of the data, contributed to the drafting and revised the manuscript critically for important intellectual content. All authors have approved the final version of the manuscript and agree to be accountable for all aspects of the work. All persons designated as authors qualify for authorship, and all those who qualify for authorship are listed.

## CONFLICT OF INTEREST STATEMENT

The authors have no conflict of interest to declare.

## Supporting information


Figure S1.

## Data Availability

The data are available from the corresponding author upon reasonable request.
