# Supplementary figures and images for: Senolytics prevent caveolar CaV3.2‐RyR axis malfunction in old vascular smooth muscle
Source: Aging Cell. 2023 Oct 14;22(11):e14002. doi: 10.1111/acel.14002 (PMC10652315; doi:10.1111/acel.14002)

Figure S1


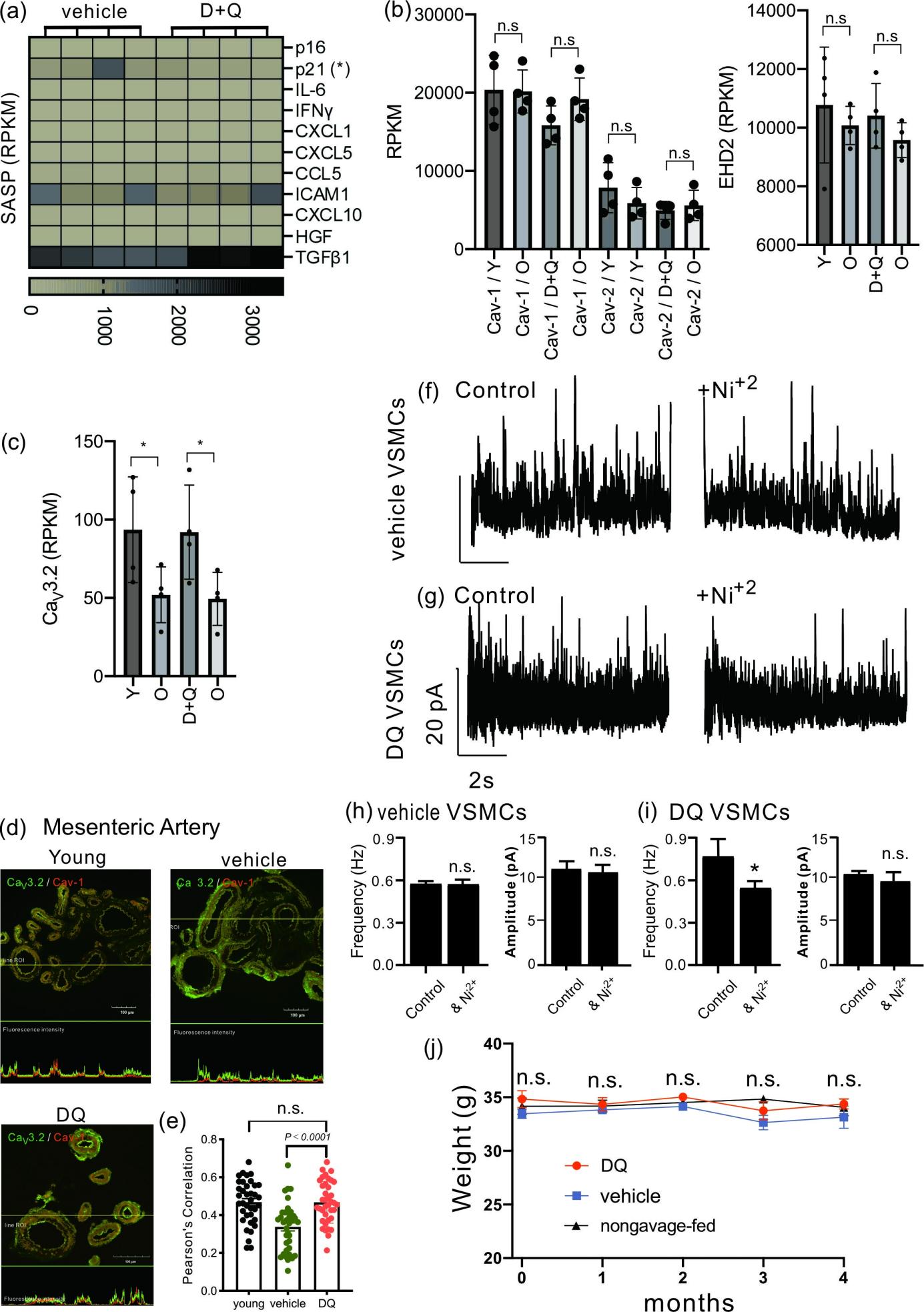


Figure S2

**
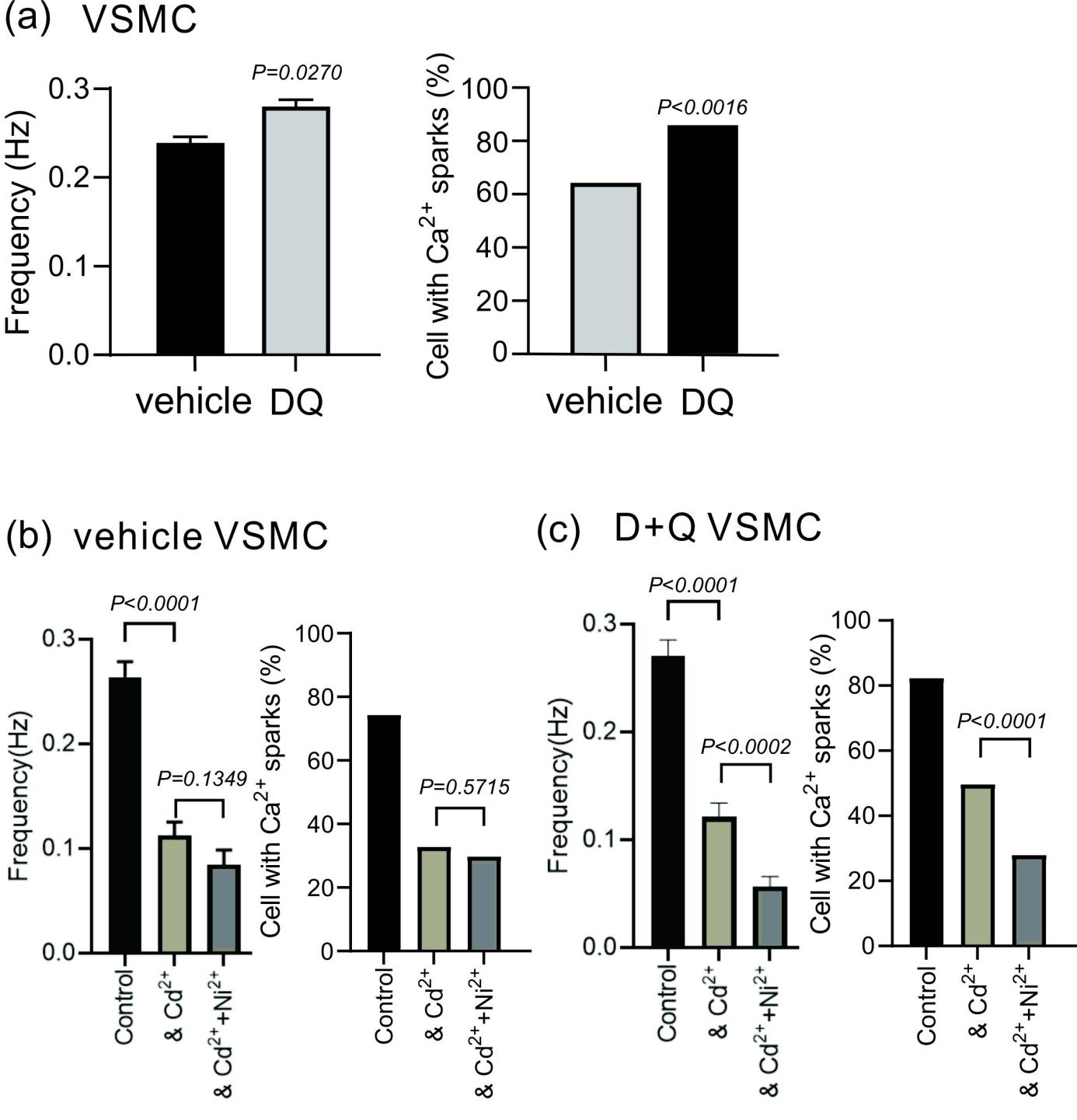
**

Supplement: Supplementary file 1 — Figure S1. [file ACEL-22-e14002-s001.docx]
